# Vasorelaxation, Induced by *Dictyota pulchella* (Dictyotaceae), a Brown Alga, Is Mediated via Inhibition of Calcium Influx in Rats

**DOI:** 10.3390/md9102075

**Published:** 2011-10-24

**Authors:** Thyago M. Queiroz, Natália T. Machado, Fabíola F. Furtado, Abrahão A. Oliveira-Filho, Maria C. Alustau, Camila S. Figueiredo, George E. C. Miranda, José M. Barbosa-Filho, Valdir A. Braga, Isac A. Medeiros

**Affiliations:** Biotechnology Center, Federal University of Paraiba, João Pessoa, PB 58.051-900, Brazil; E-Mails: thyagoqueiroz@gmail.com (T.M.Q.); naty_tabosa@msn.com (N.T.M.); fabiola.fialho@gmail.com (F.F.F.); abrahao.farm@gmail.com (A.A.O.-F); karminha@gmail.com (M.C.A); mila_farma@hotmail.com (C.S.F.); mirandag@dse.ufpb.br (G.E.C.M.); jbarbosa@ltf.ufpb.br (J.M.B.-F.); isac@ltf.ufpb.br (I.A.M.)

**Keywords:** calcium, hypotension, mesenteric artery, vasodilatation

## Abstract

This study aimed to investigate the cardiovascular effects elicited by *Dictyota pulchella*, a brown alga, using *in vivo* and *in vitro* approaches. In normotensive conscious rats, CH_2_Cl_2_/MeOH Extract (CME, 5, 10, 20 and 40 mg/kg) from *Dictyota pulchella* produced dose-dependent hypotension (−4 ± 1; −8 ± 2; −53 ± 8 and −63 ± 3 mmHg) and bradycardia (−8 ± 6; −17 ± 11; −257 ± 36 and −285 ± 27 b.p.m.). In addition, CME and Hexane/EtOAc Phase (HEP) (0.01–300 μg/mL) from *Dictyota pulchella* induced a concentration-dependent relaxation in phenylephrine (Phe, 1 μM)-pre-contracted mesenteric artery rings. The vasorelaxant effect was not modified by the removal of the vascular endothelium or pre-incubation with KCl (20 mM), tetraethylammonium (TEA, 3 mM) or tromboxane A_2_ agonist U-46619 (100 nM). Furthermore, CME and HEP reversed CaCl_2_-induced vascular contractions. These results suggest that both CME and HEP act on the voltage-operated calcium channel in order to produce vasorelaxation. In addition, CME induced vasodilatation after the vessels have been pre-contracted with L-type Ca^2+^ channel agonist (Bay K 8644, 200 nM). Taken together, our data show that CME induces hypotension and bradycardia *in vivo* and that both CME and HEP induce endothelium-independent vasodilatation *in vitro* that seems to involve the inhibition of the Ca^2+^ influx through blockade of voltage-operated calcium channels.

## 1. Introduction

Marine algae are recognized as rich sources of structurally-diverse-biologically-active compounds with great pharmaceutical and biomedical potentials. Researchers worldwide have demonstrated that marine algae derived compounds exhibit various biological activities such as anticoagulant [1,2], anti-viral [3,4], antioxidant [5,6], anti-allergic [7], anti-cancer [8,9] and as adjuvants for treating cardiopathies [10]. Despite the ascending number of new findings regarding marine algae metabolites possessing biological activity in the last three decades, few products showing real pharmaceutical potential have been identified or developed [11,12].

Marine brown algae have innumerous families, with the Dictyotaceae family being the best studied among them. The genus Dictyota is represented by more than 40 species, thus being the richest genus of the Dictyotaceae family. It is also one of the most abundant seaweeds in tropical marine habitats. The Dictyotaceae family produces a significant number of secondary metabolites, especially diterpenes [13]. Terpenoids constitute the largest family of natural products [14,15] and are classified by the homologous series of number of five carbon isoprene units in their structure: hemiterpenes C_5_ (1 isoprene unit), monoterpenes C_10_ (2 isoprene units), sesquiterpenes C_15_ (3 isoprene units), diterpenes C_20_ (4 isoprene units), triterpenes C_30_ (6 isoprene units) [16].

Many reports have extensively shown that several classes of diterpenoids exert significant cardiovascular effects [17–19]. It has also been reported that some classes of diterpenes showed significant systemic hypotensive and coronary vasodilatory effects accompanied by gradual decrease in heart rate [20,21]. The studies pointed at diterpenoids as promising sources for new prototypes in the discovery and development of novel cardiovascular therapeutic agents [19].

Cardiovascular diseases are the leading death cause in developed and developing countries [22], causing a great impact not only on human health, but also in social and economic areas. In an attempt to reduce this impact, several research groups in recent decades have worked extensively to improve the treatment of cardiovascular diseases including the discovery of new therapy strategies and drugs [23,24].

Considering that marine algae constitute great sources of bioactive compounds, the aim of this study was to investigate the mechanisms underlying the cardiovascular effects induced by the brown algae *Dictyota pulchella* in rats. To reach that goal, we employed *in vivo* and *in vitro* approaches.

## 2. Results and Discussion

### 2.1. *Dictyota pulchella* Elicits Hypotension and Bradycardia in Rats

**Figure 1:**
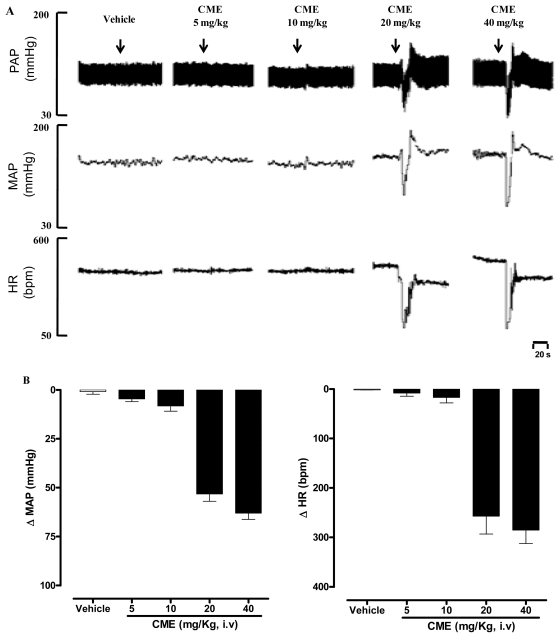
(**A**) Representative original tracings showing the changes in pulse arterial pressure (PAP, mmHg), mean arterial pressure (MAP, mmHg), and heart rate (HR, b.p.m.); (**B**) Changes in mean arterial pressure (MAP) and heart rate (HR) induced by the acute administration of increasing doses of CME (mg/kg, i.v) and vehicle. Values are expressed by mean ± SEM. (*n* = 5).

Mean arterial pressure (MAP) and heart rate (HR) were recorded before (baseline) and after intravenous administration of CH_2_Cl_2_/MeOH extract from *Dictyota pulchella* (CME, 5, 10, 20 and 40 mg/kg body weight, randomly) in conscious normotensive rats. CME elicited a dose-dependent hypotension (−4 ± 1; −8 ± 2; −53 ± 8 and −63 ± 3 mmHg) and bradycardia (−8 ± 6; −17 ± 11; −257 ± 36 and −285 ± 27 b.p.m.) as illustrated in Figure 1A,B.

It is well-known that anesthesia modifies the levels of blood pressure and heart rate interfering with central regulatory mechanisms involved in BP regulation, such as the baroreflex, by producing depression of the central nervous system synapses, altering the autonomic responses [25]. In order to avoid the possible influence of anesthesia and surgical stress on cardiovascular parameters [26], our studies were carried out in conscious freely moving rats.

In the present study, the acute administration of CME induced marked hypotension and bradycardia in conscious rats (Figure 1B). It is important to note that reduction in blood pressure due to vasodilation is usually followed by reflex tachycardia. However, under our experimental conditions, in addition to hypotension, CME induced bradycardia, which could be explained, at least in part, by a possible direct effect of the CME on the heart. Although this is an interesting possibility, this hypothesis awaits further investigation.

Although we demonstrated that CME can produce hypotension in normotensive rats, the beneficial effects of this marine algae on experimental hypertension and its clinical relevance still awaits further investigation.

### 2.2. *Dictyota pulchella* Elicits Vasorelaxation Mediated by the Blockade of Calcium Influx in Isolated Mesenteric Artery Rings

**Figure 2:**
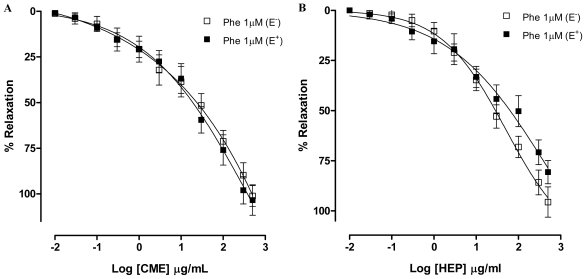
Concentration–response curves showing the relaxant effect of (**A**) CME (0.01–500 μg/mL); and (**B**) Hexane/EtOAc Phase (HEP) (0.01–500 μg/mL) on Phe (1 μM)-pre-contracted mesenteric artery rings with (■, *n* = 7) and without (□, *n* = 7) vascular endothelium, respectively. Values are expressed by mean ± SEM.

Based on the reports highlighting that the vascular smooth muscle tone plays an important role in maintaining blood pressure [27], we investigated the possible vasorelaxant effect elicited by *Dictyota pulchella* in isolated superior mesenteric arteries. An important layer in the regulation of vascular tone is the endothelium. For many years, this layer was considered to be an inert barrier that separated flowing blood from underlying tissue. Over time, it has been established that the endothelium plays an active role in regulating hemostasis, cellular and nutrient trafficking, and vasomotor tone [28]. Endothelial cells are able to produce both vasoconstrictive and vasodilating substances. The main endothelium-derived relaxing factors (EDRF) are nitric oxide, prostacyclin, and endothelium-derived hyperpolarizing factor (EDHF). Among the contracting factors are endothelin-1, thromboxane A_2_ and reactive oxygen species [29]. To evaluate the possible role played by the endothelium in the hypotension elicited by *Dictyota pulchella* observed *in vivo*, mesenteric artery rings were pre-contracted with phenylephrine (1 μM), a α_1_-adrenoceptors agonist. In the presence of this contracting agent, CME (0.01–500 μg/mL) induced a concentration-dependent relaxation (Maximum Response = 101.4 ± 4.5%; EC_50_ = 22.35 ± 5.09 μg/mL, *n* = 6) and this effect was not modified by vascular endothelium removal (Maximum Response = 103.3 ± 8.3%; EC_50_ = 21.43 ± 8.98 μg/mL, *n* = 6) (Figure 2A). Similar results were found in the presence of Hexane/EtOAc phase from *Dictyota pulchella* (HEP) (0.01–500 μg/mL), which induced a concentration-dependent vasodilatation in both intact (Maximum Response = 80.6 ± 5.8%; EC_50_ = 24.1 ± 8.95 μg/mL, *n* = 6) or denuded-endothelium (Maximum Response = 95.6 ± 7.5%; EC_50_ = 23.7 ± 5.65 μg/mL, *n* = 6) (Figure 2B).

**Figure 3:**
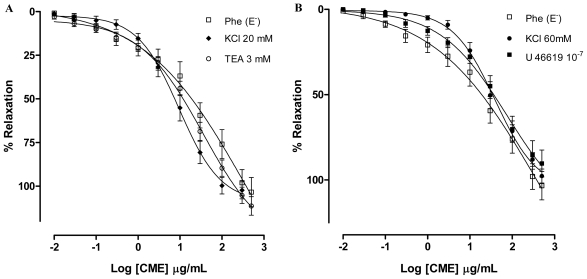

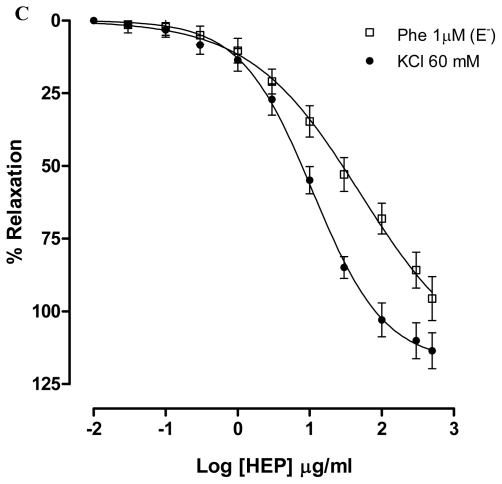
Concentration–response curves showing the relaxant effect elicited by CME (0.01–500 μg/mL, *n* = 6) in the presence of (**A**) KCl (20 mM) (♦) or TEA 3 mM (○); (**B**) KCl (60 mM) (●) or U-46619; (□) represents phenyephrine pre-contracted mesenteric rings without endothelium; (**C**) Vasorelaxant effect elicited by HEP (0.01–500 μg/mL) on KCl 60 mM-pre-contracted mesenteric artery rings (●) and Phe (1 μM)-pre-contracted mesenteric rings without endothelium (*n* = 7). Values are expressed by mean ± SEM.

Potassium channels (K^+^ channels) appear to play a crucial role in controlling the cellular membrane potential and in the vascular tone. Potassium channel openers exert their biological effects by increasing the probability of opening K^+^ channels and a number of substances that act on K^+^ channels have been shown to dilate arteries by causing hyperpolarization in vascular smooth muscle cells [30]. In addition several natural products have been shown to induce vasorelaxant effects through the activation of K^+^ channels [31–33]. Aiming to investigate involvement of potassium channels (K^+^) in the vasorelaxant activity elicited by CME, the preparations were pre-incubated with Tyrode’s modified solution containing KCl (20 mM) or with tetraethylammonium (TEA, 3 mM), a non-selective K^+^ channel blocker. In both preparations, the vasorelaxant activity was not changed (Maximum Response = 102.3 ± 4.8%; EC_50_ = 25.40 ± 6.05 μg/mL; and Maximum Response = 111.2 ± 5.3%; CE_50_ = 16.70 ± 3.61 μg/mL; *n* = 7, respectively) as shown in Figure 3A. These responses were no different from the control curve, suggesting that K^+^ channels are not involved in the vasorelaxant effect elicited by CME.

To test the hypothesis that CME produces vasorelaxant effects independent of the vasoconstrictor agent used in the preparation, mesenteric artery rings were incubated with tromboxane A_2_ agonist U-46619 (100 nM). In the presence of U-46619, CME induced concentration-dependent vasodilatation (Maximum Response = 90.3 ± 7.8%; EC_50_ = 24.63 ± 4.04 μg/mL, *n* = 6) similar to the response found by Phe-contracted mesenteric artery rings (Figure 3B).

In addition, CME produced relaxation in isolated arteries pre-contracted with KCl (60 mM). KCl depolarization elicits contraction by allowing the extracellular Ca^2+^ influx through voltage-dependent (L- and T-type) Ca^2+^ channels and subsequent calcium release from the sarcoplasmic reticulum. Due to the fact that the membrane potential is essentially clamped by the high K^+^ solution, the mechanism by which relaxation can be produced is through blockade of voltage-dependent Ca^2+^ channels, but not by other mediators that cause hyperpolarization [34]. After exposure to high concentrations of extracellular K^+^ (KCl, 60 mM), CME induced concentration-dependent vasodilatation (Maximum Response = 97.7 ± 4.0%; EC_50_ = 34.57 ± 5.11 mg/mL; *n* = 6). Under the same experimental condition, HEP induced concentration-dependent vasodilatation (Maximum Response = 113.5 ± 6.1%; EC_50_ = 10.92 ± 2.81 μg/mL; *n* = 6) (Figure 3C). Furthermore, both CME and HEP relaxed arterial segments pre-contracted with KCl (60 mM) suggesting that CME and HEP block Ca^2+^ entry through voltage-dependent Ca^2+^ channels (Ca_v_).

**Figure 4:**
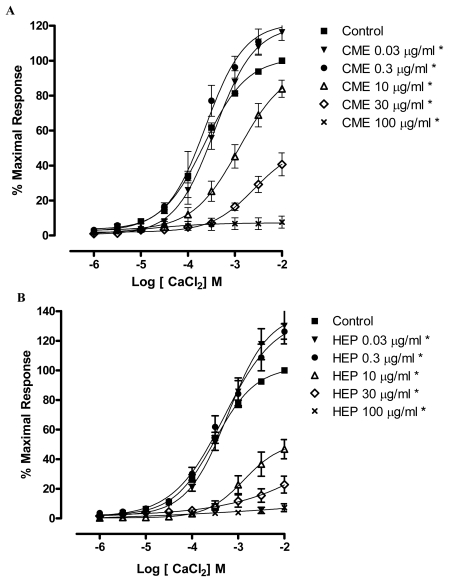
Effects of (**A**) CME and (**B**) HEP on CaCl_2_-induced contraction in endothelium-denuded mesenteric artery rings. Concentration-response curves for CaCl_2_ were determined in Ca^2+^-free solution containing KCl (60 mM). The curves were determined in the absence (control, ■) and after incubation with CME in (**A**) or HEP in (**B**) (▾) 0.03; (●) 0.3; (▵) 10; (⋄) 30; (×) 100 μg/mL, *n* = 7. * Significantly different from control (*p* < 0.05).

In order to further investigate the hypothesis that CME and HEP act through the blockade of calcium influx, CME and HEP were tested in the presence of CaCl_2_-induced contractions in a depolarizing medium without calcium. This protocol was based on the fact that CaCl_2_-induced contractions are elicited, almost exclusively, through Ca^2+^ influx, since the depolarization promoted by high concentrations of extracellular K^+^ induces the opening of voltage-dependent Ca^2+^ channels [35]. Under this experimental condition, CME and HEP (0.03; 0.3; 10; 30 and 100 μg/mL) produced a non-parallel and concentration-dependent rightward shift of the CaCl_2_ concentration–response curve significantly reducing the maximal response (Maximum Response = 116.1; 122.7; 84; 40.7; 7.6% and 130; 126.2; 46.8; 22.8; 7.3%, respectively) as illustrated in Figure 4A,B.

Voltage-dependent calcium channels (Ca_v_) are transmembrane proteins that provide influx of calcium for a variety of intracellular activities in excitable cells. In blood vessels, this calcium entry produces vasoconstriction and blockers of Ca_v_ have been used to treat cardiovascular disorders. Voltage-dependent calcium channels consist of different subunits: the α_1_-subunit, which contains four homologous transmembrane domains encompassing the pore, the voltage sensor and the selectivity filter; and the β, α2δ and γ auxiliary subunits. The α_1_-subunits have been classified as Ca_v_1.1, Ca_v_1.2, Ca_v_1.3, Ca_v_1.4 (L-type Ca_v_), Ca_v_2.1 (P/Q-type Ca_v_), Ca_v_2.2 (N-type Ca_v_), Ca_v_2.3 (R-type Ca_v_), Ca_v_3.1, Ca_v_3.2 and Ca_v_3.3 (T-type Ca_v_). In the vascular smooth muscle cells two types are expressed: L-type and T-type. The first ones are more expressed in these cells and exert an important role in the regulation of the vascular tone [36,37].

**Figure 5:**
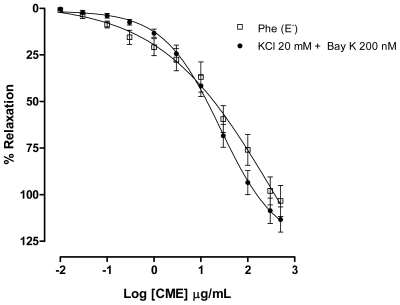
Effect of increasing concentrations of CME (0.01–500 μg/mL, *n* = 7) on phenylephrine (Phe, 1 μM) (□) or KCl (20 mM) plus S(−)-BayK 8644 (10^−7^ M) (▴) induced contractions in isolated mesenteric artery rings without endothelium. Values are expressed by mean ± SEM.

In order to examine which subtype of Ca_v_ was involved in the vasorelaxant effect elicited by CME, a contraction with Bay K 8644 (200 nM), a L-type Ca^2+^ channel agonist, was evoked. CME induced concentration-dependent vasodilatation (Maximum Response = 113.3 ± 6.7%; EC_50_ = 19.45 ± 6.66 μg/mL, *n* = 7) and it was similar to the response found under Phe-induced contractions (Figure 5). These data indicate that L-type Ca_v_ channels could be involved in the vasorelaxant effect induced by CME.

**Figure 6:**
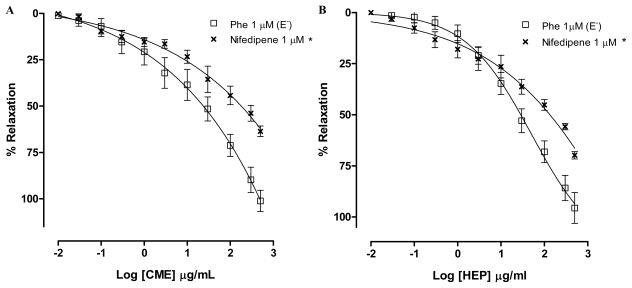
Concentration–response curves showing the relaxant effect elicited by (**A**) CME (0.01–500 μg/mL); and (**B**) HEP (0.01–500 μg/mL) in the presence of nifedipine (×, *n* = 6); (□) represents phenyephrine pre-contracted mesenteric rings without endothelium. Values are expressed by mean ± SEM. * Significantly different from control (*p* < 0.05).

In order to investigate a role for dihydropyridine calcium channels, rings were incubated with nifedipine. In the presence of nifedipine, CME (0.01–500 μg/mL) induced a concentration-dependent relaxation (Maximum Response = 63.6 ± 2.9%; EC_50_ = 25.4 ± 11.0 μg/mL, *n* = 6). The maximum response was different to the response found by Phe-contracted mesenteric artery rings (Figure 6A). In addition, in the presence of nifedipine, HEP (0.01–500 μg/mL) induced a concentration-dependent relaxation (Maximum Response = 69.8 ± 1.7%; EC_50_ = 24.4 ± 10.3 μg/mL, *n* = 6). The maximum response was different to the response found by Phe-contracted mesenteric artery rings (Figure 6B). Based on these findings, it can be suggested that the endothelium-independent vasodilatation induced by both CME and HEP involves, at least in part, the inhibition of the Ca^2+^ influx through blockade of calcium channels.

## 3. Experimental Section

### 3.1. Algal Material and Preparation of CH_2_Cl_2_/MeOH Extract (CME) and Hexane/EtOAc Phase (HEP) from *Dictyota pulchella*

The brown alga *Dictyota pulchella* Hörnig and Schnetter (Dictyotaceae, Phaeophyceae) was collected at Bessa Beach, João Pessoa, State of Paraíba, Brazil, at coordinates 07°04′01″S and 34°49′35″W in February 2009 at a depth of 1 m and it was identified by George Emmanuel C. de Miranda. A voucher specimen (JPB 41771) is deposited at Lauro Pires Xavier Herbarium of the Federal University of Paraíba.

The freeze-dried material (240 g) was extracted with CH_2_Cl_2_/MeOH (2:1) at room temperature. The concentrated extract (10 g) was partitioned by vacuum filtration with silica gel that uses the solvents Hexano/EtOAc (9:1) to give the fraction Hexano/EtOAc (9:1) (850 mg).

The CH_2_Cl_2_/MeOH extract (CME) and Hexane/EtOAc phase (HEP) were prepared in a mixture of distilled water (*in vitro* experiments) or NaCl 0.9 % solution (*in vivo* experiments) and cremophor (0.013% v/v) and kept at −4° C. The stock solution was diluted to the desired concentrations with distilled water or isotonic saline just before use. The final concentration of cremophor in the bath had no effect when tested in control preparations (data not shown).

### 3.2. Drugs and Solutions

The following drugs were used: Cremophor EL, l-phenylephrine chloride (Phe), acetylcholine chloride (Ach), tetraethylammonium (TEA), S(−)-Bay K 8644, sodium nitroprusside, Ethylene glycol-bis(2-aminoethylether)-*N*,*N*,*N′*,*N′*-tetraacetic acid (EGTA) and 9,11-Dideoxy-11α,9α-epoxymethanoprostaglandin F_2α_ (U 46619), were purchased from Sigma Chemical (Sigma Chemical Co., St. Louis, MO, USA). Heparin sodium salt (Roche Brazil, São Paulo, Brazil), sodium thiopental (Cristália, São Paulo, SP, Brazil). The substances were dissolved in distilled water (*in vitro* experiments) or in NaCl 0.9% solution (*in vivo* experiments).

The composition of the Tyrode’s solution used to bath isolated rings was (mM): NaCl, 158.3; KCl, 4.0; CaCl_2_, 2.0; MgCl_2_, 1.05; NaH_2_PO_4_, 0.42; NaHCO_3_, 10.0 and glucose, 5.6.

### 3.3. Animals

Male Wistar rats (250–300 g) were used in all experiments. They were housed in conditions of controlled temperature (21 ± 1 °C) and exposed to a 12 h light-dark cycle with free access to food (Labina^®^, PURINA, Brazil) and tap water. All procedures described in the present study are in agreement with Institutional Animal Care and Use Committee of the Federal University of Paraiba (CEPA/LTF protocol No. 0208/10).

### 3.4. *In Vivo* Experiments

Intra-aortic blood pressure was recorded using a technique described by Braga [37] under sodium thiopental anesthesia (45 mg/kg, i.v.), the lower abdominal aorta and inferior vena cava were cannulated via left femoral artery and vein using polyethylene catheters. Thereafter, catheters were filled with heparinized saline solution and tunneled under the skin to emerge between the scapulae. Arterial pressure was measured 24 h after surgery by connecting the arterial catheter to a pre-calibrated pressure transducer (MLT0380/D, ADInstruments, Australia) and connected to a computer (Mikro-tip Blood pressure system, ADInstruments, Australia) running the LabChart software (ADInstruments, Australia). The data were sampled at 2000 Hz. For each pulse pressure, the computer calculated mean arterial pressure (MAP) and heart rate (HR). The venous catheter was used for drug administration.

### 3.5. *In Vitro* Experiments

#### 3.5.1. Tissue Preparation

Rats were euthanized by stunning and bleeding. The superior mesenteric artery was removed and cleaned from connective tissue and fat. Rings (1–2 mm) were obtained and placed in physiological Tyrode’s solution, maintained at 37 °C, gassed with carbogenic mixture (95% O_2_ and 5% CO_2_), and maintained at pH 7.4. All preparations were stabilized under a resting tension of 0.75 g for 1 h. The solution was replaced every 15 min to prevent the accumulation of metabolites. The force of contraction was isometrically recorded by a force transducer (MLT020, ADInstruments, Australia) connected to a data acquisition system (ML870/P with LabChart version 7.0, ADInstruments, Australia). Endothelium was removed by gently rubbing the intimal surface of the vessels.

The presence of functional endothelium was assessed by the ability of acetylcholine (ACh) (10 μM) to induce more than 90% relaxation of pre-contracted vessels with phenylephrine (10 μM). When the relaxation to ACh was less than 10%, this was taken as evidence that the vessel segments were functionally denuded of endothelium.

#### 3.5.2. Measurement of Vascular Relaxation Elicited by CME or HEP

The ability of extract or phase to cause vascular relaxation was evaluated in both endothelium-intact and endothelium-denuded mesenteric artery rings previously contracted by Phe (1 μM). Under the sustained contraction elicited by Phe the vessels were exposed to cumulative concentrations of CME or HEP (3, 5, 10, 30 and 50 μg/mL).

#### 3.5.3. Effect of CME or HEP on KCl (60 mM) or U-46619 (100 nM)-Induced Contractions in Rings without Endothelium or after KCl (20 mM) and Tetraethylamonium (3 mM) Incubation

Contractions of the vessels were induced with KCl (60 mM) or U-46619 (100 nM) in rings without the endothelium. During the tonic phase of the contraction, CME or HEP (0.01–500 μg/mL) was added to the organ bath. The extent of relaxation was expressed as the percentage of KCl- or U46619-induced contraction. Furthermore, curves for CME were obtained after incubation with KCl (20 mM) or TEA (3 mM), a non-selective K^+^ channel blocker in rings without the endothelium.

#### 3.5.4. Depolarization Induced by High Extracellular K^+^ Concentration

In order to access the effects of CME or HEP on voltage-gated Ca^2+^ channels, superior mesenteric artery rings were bathed for 15 min in Ca^2+^-free Tyrode’s solution, prepared by omitting only CaCl_2_ and then exposed for an additional 15 min to a high K^+^ (60 mM) Ca^2+^-free solution. Under this new experimental condition, cumulative concentration-response curves to CaCl_2_ (ranging from 1 μM to 3 mM) were obtained. CME and HEP (0.01–500 μg/mL) were added to the preparations for 30 min, and then a new cumulative concentration–response curve for CaCl_2_ was determined. The maximal contraction obtained with the control concentration–response curve to CaCl_2_ was taken as 100% and all values were calculated as a percentage of the maximal response. Each preparation was exposed to only one CME or HEP-concentration. All experiments were done using endothelium-denuded superior mesenteric artery rings.

#### 3.5.5. Effect of CME on the Contraction Elicited by S(−)-Bay K 8644 (200 nM) or Nifedipine (1 μM) in Mesenteric Artery Rings without Endothelium

Contractions of the vessels were induced by S(−)-Bay K 8644 (200 nM), an activator of calcium channels sensitive to dihydropyridines, in rings without the endothelium. During the tonic phase of the contraction, CME (0.01–500 μg/mL) was added to the organ bath. The extent of relaxation was expressed as the percentage of S(−)-Bay K 8644 -induced contraction. In addition, in order to intestigate the involvement of calcium channels, nifedipine (1 μM) was used to pre-incubate the preparations prior to CME or HEP administration.

### 3.6. Statistical Analysis

Data are expressed as the mean ± SEM. When appropriate, the significance of differences was determined using one-way ANOVA following Bonferroni’s post test with GraphPad Prism version 5.0 (GraphPad Software, La Jolla, CA, USA). Throughout the results, the “maximal relaxation” corresponds to the maximum response of pre-contracted tissues to the highest concentration of drug tested. *P* < 0.05 was considered significant.

## 4. Conclusions

Using combined *in vivo* and *in vitro* approaches, our data suggest that *Dictyota pulchella* induces hypotension and bradycardia. In addition, both extract and phase from *Dictyota pulchella* induce endothelium-independent vasodilatation that involves the inhibition of the Ca^2+^ influx through blockade of voltage-operated calcium channels. More studies are needed in order to evaluate the effects of this marine alga on experimental models of diseases such as hypertension and heart failure, which will help to advance the field towards clinical trials. Eventually, these data will open new perspectives in the use of these marine brown algae for developing drugs targeting the cardiovascular system.
